# Epigenetic regulation of adult neural stem cells: implications for Alzheimer’s disease

**DOI:** 10.1186/1750-1326-9-25

**Published:** 2014-06-25

**Authors:** Carlos P Fitzsimons, Emma van Bodegraven, Marijn Schouten, Roy Lardenoije, Konstantinos Kompotis, Gunter Kenis, Mark van den Hurk, Marco P Boks, Caroline Biojone, Samia Joca, Harry WM Steinbusch, Katie Lunnon, Diego F Mastroeni, Jonathan Mill, Paul J Lucassen, Paul D Coleman, Daniel LA van den Hove, Bart PF Rutten

**Affiliations:** 1 Center for Neuroscience, Swammerdam Institute for Life Sciences, University of Amsterdam, SciencePark 904, 1098XH Amsterdam, The Netherlands; 2 Department of Translational Neuroscience, School of Mental Health and Neuroscience (MHENS), Maastricht University, Maastricht, the Netherlands; 3 Department Psychiatry, Brain Center Rudolf Magnus, University Medical Center Utrecht, Utrecht, The Netherlands; 4 School of Pharmaceutical Sciences of Ribeirão Preto, University of São Paulo, São Paulo, Brazil; 5 University of Exeter Medical School, RILD Level 4, Barrack Road, University of Exeter, Devon, UK; 6 Department of Psychiatry and Neuropsychology, School for Mental Health and Neuroscience, Maastricht University Medical Centre, P.O. Box 616, 6200 MD Maastricht, The Netherlands

**Keywords:** Adult neurogenesis, Epigenetics, Alzheimer’s disease, DNA methylation, Histone modifications, MicroRNAs, Stem cell, Induced pluripotent stem cell

## Abstract

Experimental evidence has demonstrated that several aspects of adult neural stem cells (NSCs), including their quiescence, proliferation, fate specification and differentiation, are regulated by epigenetic mechanisms. These control the expression of specific sets of genes, often including those encoding for small non-coding RNAs, indicating a complex interplay between various epigenetic factors and cellular functions.

Previous studies had indicated that in addition to the neuropathology in Alzheimer’s disease (AD), plasticity-related changes are observed in brain areas with ongoing neurogenesis, like the hippocampus and subventricular zone. Given the role of stem cells e.g. in hippocampal functions like cognition, and given their potential for brain repair, we here review the epigenetic mechanisms relevant for NSCs and AD etiology. Understanding the molecular mechanisms involved in the epigenetic regulation of adult NSCs will advance our knowledge on the role of adult neurogenesis in degeneration and possibly regeneration in the AD brain.

## Introduction

Neural stem cells (NSCs) are present in some areas of the adult brain that continue to produce new, functional neurons that are added to existing brain circuits. So-called neurogenic ‘niches’ are unique tissue microenvironments that are permissive to the presence of NSCs in the adult brain [1]. These have now been identified and characterized in the subgranular zone (SGZ) of the hippocampal dentate gyrus and in the subventricular zone (SVZ) of the lateral ventricles. In these regions, new neurons are produced from NSCs throughout life in several species including human [2,3]. The NSC niches may differ strongly from other stem cell niches, e.g. in the periphery, not only because no neurons are generated there, but also because the local context, tissue matrix, somatic support cell population (micro-/astroglia), vasculature, adhesion molecules, growth factors, metabolism etc, are specifically composed in different adult tissue stem cell microenvironments, providing a “homing” environment for stem cells [4-14]. Perhaps more relevant in the context of this review, niches may differ considerably even within the central nervous system and may, for instance, influence NSC fate, as stem cells harvested from the spinal cord, normally destined to form motor neurons with axons spanning long distances, form small granule neurons when transplanted into the hippocampus [7]. Therefore, although it is currently accepted that a combination of niche signals and cell-intrinsic programs orchestrate the transition from an undifferentiated NSC state to a progenitor cell committed to the neuronal fate [15], epigenetic mechanism such as miRNAs may play a role in this regulation [16]. This discussion seems relevant because, specific (NSC) niche characteristic may condition intrinsic vulnerability of different stem cells, and specific niche supplementation could be a viable strategy to support proliferation, differentiation or self-renewal [17,18], however, an extensive discussion of this topic escapes the aim of this review.

The process of adult neurogenesis is also regulated, e.g. by environmental and hormonal factors like stress, growth factors, exercise and antidepressant drugs while alterations have also been observed in neurodegenerative disorders [19], like epilepsy, stroke [20] or Alzheimer’s disease (AD), that suggested neurogenesis responds to these conditions [21]. However, although the neurogenic capacity in disorders like AD clearly is insufficient or inappropriate to compensate for the neuronal dysfunction or loss [22,23], stimulation of the molecular pathways that regulate adult neurogenesis may be an attractive therapeutic or preventative target to boost the brain’s regenerative capacity.

Multiple intrinsic and extrinsic factors have been identified, such as growth factors, morphogens, transcription factors and cell cycle regulators, which control NSC maintenance in the adult neurogenic niche and their differentiation into mature neurons. However, none of them act in isolation and most function in networks of signaling molecules that influence each other [15,24,25]. Epigenetic mechanisms are likely key players within these signaling networks, as DNA methylation, chromatin remodeling and small non-coding RNAs from the microRNAs superfamily are required for the fine-tuning and coordination of gene expression during adult neurogenesis [16]. The aim of the present manuscript is to review the involvement and relevance of epigenetic regulation in adult NSCs and to discuss their possible role in regulating adult neurogenesis under conditions of neurodegeneration and AD.

## Adult neurogenesis

The maintenance and development of adult NSCs in the SVZ and SGZ occurs within a highly specialized microenvironment in which these cells reside, known as the neurogenic ‘niche’ [26] in which a variety of other cell types reside as well, including endothelial cells, astrocytes, microglia, NSC progeny and mature neurons, that are all part of the microenvironment of the neurogenic niche and may contribute to the development of new neurons. In addition, several extrinsic and intrinsic signaling molecules regulate neurogenesis in these areas while cells outside the neurogenic niche might also be of influence through their connections with, and projections to, cells within the neurogenic niche. The unique microenvironment of the neurogenic niche is thought to allow NSCs to proliferate, differentiate, migrate, maturate and integrate in the existing, mature neural network [15,26,27].

The SGZ and SVZ (or subependymal zone [SEZ]) of the adult mammalian brain contain different types of NSCs that give rise to mature neurons. In the SVZ, the primary NSCs are slow proliferative radial glia-like cells (type B cells) that express the astrocytic marker glial fibrillary acidic protein (GFAP). These cells may serve as the quiescent NSCs of the SVZ and give rise to rapidly proliferating transit-amplifying progenitor cells (type C cells). Also splice variants of the GFAP gene, i.e. the GFAP delta isoform have been associated with stem cells in this region in rodent and human brain. The type C cell is seen as an intermediate cell type that generates neuroblasts (type A cells) that express the neuronal marker doublecortin (DCX) [28,29]. These cells are able to migrate from the SVZ through the rostral migratory stream (RMS) to the olfactory bulb (OB) where they primarily differentiate into GABAergic interneurons. A minority of the neuroblasts differentiate into dopaminergic interneurons [3]. The stem cell marker Nestin is expressed by type A, B and C cells. A fourth cell type lining the lateral ventricles was found to also express Nestin. These ependymal cells, or type E cells, exhibit some neural stem cell characteristics [28].

In the hippocampal SGZ, putative NSCs have also been identified. Type 1 hippocampal progenitor cells are radial glia-like cells that express GFAP, similar to the SVZ type B cells. In addition, they express the stem cell marker Nestin and the sex determining region Y (SRY)-box 2 (Sox2) transcription factor. These cells may represent a quiescent/slow proliferative neural stem cell pool and are able to produce cells from at least the astrocytic and neural progeny. Type 2a cells are proliferative non-radial hippocampal progenitor cells that, in general, do not express GFAP. Type 2a cells are actively proliferating and generate other transient neuroblasts with some different characteristics known as Type 2b and Type 3 cells, and their commitment to the neural fate is marked by the expression of immature neuron markers like DCX and polysialic acid neural cell adhesion molecule (PSA-NCAM) [30]. These cells migrate into the inner granule cell layer (GCL) of the dentate gyrus (DG), where they start to express calretinin and the granule cell marker Prox-1 before they differentiate into glutamatergic granule cells and integrate into the existing hippocampal neuronal network [24,31].

The important role of the specific microenvironment in the neurogenic niches for the regulation of NSCs in the SGZ and SVZ has been shown by several *in vivo* and *in vitro* studies. For example, rat glial progenitor cells can change to a neuronal fate when transplanted into a neurogenic region [7], while mouse SVZ neural progenitors committed to the neuronal lineage, changed to glial differentiation upon transplantation into regions outside the neurogenic niche [32]. Moreover, neuroblasts can change their fate and differentiate into oligodendrocytes upon a change in the microenvironment induced by demyelination of the corpus callosum [33].

The microenvironment of the neurogenic niche is composed of, and maintained by, several components, including local cell types, cell signals from more distal sources, the extracellular matrix and the microvasculature. Of these, the microvasculature has been argued to be one of the most important structures in maintaining the functional role of the neurogenic niche [10,34-37], especially in setting the balance between proliferation and quiescence of NSCs. Indeed, the SVZ and SGZ appear to be highly vascularized by a dense network of specialized capillaries [38]. It has been shown *in vitro* that endothelial cells (ECs) can stimulate NSC self-renewal and neurogenesis through secreted soluble factors [35,39,40] and that NSCs closely interact with the microvasculature [10,34,36,41]. Additionally, the blood flow and hemodynamics of this intricate network affect NSC proliferation and can also act as a scaffold during migration [34,42-46]. In addition, microglia, the brain resident macrophages, have a significant role in the regulation and maintenance of neurogenesis in the SGZ [47]. Importantly, microglia may inhibit the proliferation of neural stem/progenitor cells despite the absence of inflammatory stimulus [48]. Thus, in addition to fate determination and cell differentiation, the microenvironment of the neurogenic niche is important for self-renewal, proliferation, migration and maturation of NSCs. The exact mechanisms that regulate these processes within the adult neurogenic niches are now starting to be identified and interestingly, many of the mechanisms regulating neurogenesis during embryonic development, appear to be conserved in adulthood, and both intrinsic and extrinsic factors important for embryonic neurogenesis, including epigenetic regulation, are also involved in the regulation of neurogenesis in the adult brain [24].

## Epigenetic mechanisms in NSCs

Although the definition of epigenetics is broad and has been modified over the years, it is currently generally accepted to refer to changes in gene activity independent of the primary DNA sequence. In some definitions, only the modification of activity states inherited across cell division is considered, consistent with an important role in the regulation of proliferative cells in the brain [49]. Thus, independently of the genotype, different epigenetic profiles may result in different phenotypes. Mechanisms like DNA (hydroxy)methylation, histone tail modifications and regulation by non-coding RNAs are responsible for these alterations [50]. Alterations in gene expression patterns induced by these mechanisms may be more frequent than changes in the hard-coded genetic information, such as genetic mutations. Moreover, also environmental influences can induce epigenetic modifications and exert long-lasting effects throughout the life-span of an organism. In addition, many of these epigenetic modifications are heritable through mitoses and transgenerational effects have been reported too [16,51-53].

Epigenetic mechanisms play a key role in cell type specification and the development of most tissues. Consistent with this concept, adult neurogenesis is under intensive regulation by epigenetic mechanisms [16] and both temporal and spatial control of gene expression is executed by epigenetic mechanisms together with other signaling molecules. This is essential for the regulation of the sequential stages of neurogenesis. Intrinsic control of neurogenesis by epigenetic mechanisms within NSCs, and extrinsic control through epigenetic regulation of gene expression within non-NSC cells, which form part of the neurogenic niche, likely contributes to the maintenance of a continuous supply of new neurons in the adult brain [16,51-53]. In particular, epigenetic modifications are capable of controlling (transient) gene repression that are necessary for NSC pluripotency and proliferation. Furthermore, NSC fate is determined in part by the permanent silencing of specific genes through epigenetic mechanisms. Upon cell fate determination, repression of NSC differentiation-related genes is e.g. removed and a permanent repression of the non-cell lineage specific genes is induced. Hence, expression of cell lineage-specific genes is increased and NSC cell differentiation is initiated. Individual mechanisms of DNA and histone modifications and non-coding RNAs are responsible for these changes in gene expression patterns. In addition, these mechanisms interact and are capable of influencing each other, forming a complex network of epigenetic and non-epigenetic regulation of adult neurogenesis [16,53,54]. Several epigenetic mechanisms that control self-renewal and differentiation of NSCs have been identified and will be discussed below.

### DNA methylation

DNA methylation is one of the most common epigenetic mechanisms and refers to the addition of a methyl group to the carbon 5 position of the pyrimidine ring of the DNA base cysteine, which results in the generation of 5-methylcytosine (5-mC). DNA methylation is specifically high at CpG islands and usually results in gene repression. DNA methyltransferases (DNMT) are the enzymes that catalyze the reaction of DNA methylation. DNA methyltransferase 1 (DNMT1) is e.g. important for the maintenance of DNA methylation over multiple cell divisions. After DNA replication, the hemi-methylated DNA is recognized by DNMT1 and methylation of the non-methylated DNA strand is induced. DNMT1 thus maintains DNA methylation through mitoses and is responsible for the heritability of the DNA methylation marks. DNMT3a and DNMT3b on the other hand induce *de novo* DNA methylation at former un-methylated DNA [52]. Whereas these processes have long been regarded as strictly separate, emerging evidence suggests that these processes overlap far more. Localization of DNMT1 at the replication fork and its role in DNA methylation repair suggest a role in replication of DNA with methylation marks, whereas DNMT3a likely has a role in DNA methylation repair, similar to its role in prokaryotes [55,56]. Interestingly, recent studies have pointed to a role for non-CpG methylation, possibly mediated by DNMT3a, in embryonic stem cells [57].

DNA methylation and demethylation are dynamic processes and thought to translate changes in the environment to changes in gene expression. Recent literature has highlighted the links between environmental influences during development/early life, such as temperature, toxic chemicals, nutrition, tobacco smoke and alcohol and their consequences on DNA methylation and other epigenetic mechanisms [58,59]. In turn, DNA methylation directly and indirectly influences gene expression. Prevention of transcription factor binding by methylation at gene target sequences has a direct effect on gene expression. Indirectly, the binding affinity of other transcriptional regulators, including co-activator and co-repressor factors and complexes is modulated by DNA methylation. Together, *de novo* methylation and maintenance of methylation marks, either directly or indirectly affecting gene expression, are capable of regulating sequential steps of adult neurogenesis [51,54].

#### ***DNA methylation and adult NSCs***

Recent observations have suggested that epigenetic mechanisms could be sensors of environmental changes and fine modulators of adult hippocampal neurogenesis [60]. Enrichment of the environment, a well-known stimulus of hippocampal neurogenesis, to which exercise contributes the most, could promote neuronal maturation, possibly through increased methylation activity [59,60]. In addition, alterations in neurogenesis associated with pathological conditions of the brain have been linked to changes in DNA methylation in the brain [60]. The possible mechanisms by which DNA methylation could influence different stages of adult neural stem cells in both the SGZ and the SVZ will be discussed.

*In vitro* analysis of DNMT function in differentiating NSCs has proven to be a relevant experimental approach to study the role of DNMTs and DNA methylation in neurogenesis [61]. Neurosphere formation and inhibition of differentiation of cultured quiescent NSCs was maintained by application of epidermal growth factor (EGF) and fibroblast growth factor (FGF). Withdrawal from EGF/FGF supplementation induced their differentiation and subsequent immunostaining confirmed DNMT1 and DNMT3a expression and presence of DNA methylation in undifferentiated NSCs. At the start of differentiation, DNMT1 and DNMT3a were increased but subsequently decreased upon migration and their late differentiation. Thus, while high expression was observed in undifferentiated cells, DNMT1 and DNMT3a expression decreases in the differentiating/migrating NSCs. Importantly, Chromatin immunoprecipitation (ChIP) analysis showed that both increases and decreases in methylation occur in differentiating NSCs at different loci [61]. This possibly reflects a combined repression of stem cell maintenance genes and an activation of cell differentiation genes. Nonetheless, a role for DNA methylation in NSC differentiation and migration is further supported by data showing that administration of the methylation blocker 5-azacytidine (AZA) decreases NSC differentiation and migration [61].

The role of DNMT3a in neuronal differentiation has been further confirmed in the mouse postnatal brain. Wu and colleagues [62] observed expression of DNMT3a in both the SVZ and SGZ in the postnatal mouse, while a more detailed immunohistochemical study found two distinct types of DNMT3a-immunoreactive cells in the SGZ. The first type of immunoreactive cells (those with relatively low immunoreactivity) is ubiquitously expressed throughout the hippocampus, while the second type (displaying high levels of immunoreactivity) was particularly found in the neurogenic region of the SGZ [63]. Immunohistochemical analyses 3 weeks after 5-bromo-2′-deoxyuridine (BrdU) administration showed that the high expressing DNMT3a cells in the SGZ were newborn and expressed the mature neuron marker NeuN. In agreement with this observation, knockout of DNMT3a *in vivo* results in a profound decrease in postnatal neurogenesis in both the SVZ and SGZ [63]. Culturing NSCs from DNMT3a knockout mice confirmed that DNMT3a is necessary for neuronal differentiation. A 10-fold decrease in newborn neurons upon differentiation induction was observed in DNMT3 knockout NSCs, again indicative of impaired neurogenesis [62]. ChIP analysis revealed that the DNMT3a targets were enriched among the differentially expressed genes in NSCs obtained from DNMT3 knockout mice. Moreover, the down-regulated genes in DNMT3a knockout mice were neurogenic genes while the up-regulated genes were genes involved in astroglial and oligodendroglial differentiation [62]. Thus, DNMT3a seems to act in NSCs as a switch that regulates gene expression towards the non-neuronal lineage when downregulated, and towards a neurogenic fate when upregulated.

Indirect regulation of gene expression by DNMTs is mediated through proteins with methyl-CpG-binding domains (MBDs). MBDs bind to methylated gene promoters, thereby inhibiting gene expression through blockage of transcription factor binding or recruitment of other enzymes that induce transcriptional repression [51]. Similar to the DNMT expression changes described in the previous paragraph, expression of MBD1 correlates with neuronal differentiation [61]. Accordingly, low MBD1 expression was found in undifferentiated neurospheres. Although a moderate increase in expression levels was observed at the induction of differentiation, subsequent down-regulation was seen upon the start of the migratory phase. This suggests that MBD1 target genes are highly expressed in the self-renewing NSCs due to low levels of MBD1 expression. Then, increased MBD1 expression leads to repression of these genes, allowing cell differentiation [61]. Since MBD1 expression is predominantly found in neurons of the adult brain, MBD1 seems to have a specific role in inducing or maintaining neuronal differentiation. Indeed, MBD1-deficient mice have reduced neurogenesis in the postnatal but not embryonic brain [64]. BrdU analysis showed that although there were no differences at day 1, the amount of BrdU labeled cells in the MBD1-deficient mice was significantly decreased 4 weeks after the BrdU injection. This was accompanied by impaired neurogenesis and lower cell density in the DG of the hippocampus. Subsequent phenotypical analysis of the surviving newborn (BrdU labeled) cells revealed that in addition to the overall decrease in BrdU-labeled cells, newborn neurons were significantly more affected than other, more immature, phenotypes. Additionally, the percentage of newborn astrocytes was increased [64]. Thus, MBD1 may be important for neuronal differentiation of NSCs and survival of newborn neurons in the postnatal brain.

The role of MBD1 in adult neurogenesis and NSC differentiation was confirmed by Li and colleagues [65] who provided additional information on the molecular mechanism involved. NSCs isolated from adult MBD1 knockout mice showed increased fibroblast growth factor 2 (FGF2) expression. Moreover, overexpression of MBD1 in both MBD1 knockout and wild type NSCs decreased FGF2 expression. *In vitro* ChIP analysis confirmed the specific binding of MBD1 to the FGF2 promoter while hypomethylation of the FGF2 promoter in MBD1 knockout mice was observed [66]. Importantly, all the events that either led to a decrease in MBD1 expression or an increase in FGF2 expression resulted in reduced neuronal differentiation [65]. This suggests that neuronal differentiation in the postnatal and adult brain is dependent on methylation of, and MBD1 binding to, the FGF2 promoter, which results in its repression.

A second member of the MBD protein family, methyl-CpG-binding protein 2 (MeCP2) regulates gene expression through a similar mechanism as MBD1. It binds to methylated DNA and functions as a transcriptional repressor. Although MeCP2 expression is predominantly found in neurons, immunohistochemistry on MeCP2 knockout mice brains indicated a different additional function [67]. Although no difference in the amount of newborn neurons was observed in MeCP2 knockout mice, dendritic spine formation and spine density were decreased, resulting in delayed and impaired maturation of the newborn neurons. This was accompanied by a decreased expression of genes important for synaptogenesis [67]. Together, it suggests that, in contrast to a role for MBD1 in early neurogenesis, MeCP2 binding to DNA methylation marks is important for regulating the expression of genes involved in maturation of newborn neurons.

MeCP2 may also function to repress non-neuronal lineage genes and maintain neuronal identity, allowing proper neuronal differentiation. Kohyama and colleagues [68] found high expression of MeCP2 in mature hippocampal neurons of the adult mouse brain. Subsequent analysis of the DNA methylation status of different hippocampal cell types revealed high levels of methylation around the transcriptional start region of the GFAP gene. Moreover, MeCP2 expression was absent in oligodendrocytes and astrocytes in the hippocampus [68]. Thus, also repression of GFAP expression by binding of MeCP2 to methylated DNA loci is important for newborn neuron maturation. Further support for a role for MeCP2 in maintaining neuronal cell fate was shown by *in vivo* transplantation of MeCP2-expressing neural progenitor cells in non-neurogenic regions [69]. MeCP2 expression allows neuronal differentiation in these areas where usually astrocytic differentiation is observed. Moreover, expression of a truncated mutant form of MeCP2, lacking essential domains of the wild type MeCP2, did not allow NSC neuronal differentiation under astrocytic differentiation-inducing conditions, indicating that MeCP2 binding to methylated DNA is a key regulatory factor of this process [69]. Thus, although MeCP2 may not regulate the initiation of NSC differentiation, it may be important for neuronal differentiation and neuronal cell fate. Furthermore, while MeCP2 is not required for the production of immature neurons in the DG, the newly generated neurons, in the absence of MeCP2, exhibit pronounced deficits in neuronal maturation, including a delayed transition into a more mature stage, altered expression of presynaptic proteins and reduced dendritic spine density, suggesting that MeCP2 plays a role in other aspects of neuronal maturation, including dendritic development and synaptogenesis [67].

Early studies identified mutations in MeCP2 that cause neurodevelopmental alterations accounting for the majority of Rett syndrome cases and more recent studies suggest that MeCP2 plays an important role in brain development, aging and in neurological disorders [70]. The extreme abundance of MeCP2 expression in the brain, estimated extend to one molecule of MeCP2 for every two nucleosomes in neuronal chromatin [71] suggesting that it may play a key role in neurological disorders associated with aberrant DNA methylation, such as AD. Particularly in the case of Rett Syndrome, the most common genetic cause of severe intellectual disability in females, several studies in animal models of the disease have demonstrated that animal do not develop an irreversible condition and that phenotypic rescue may be possible, highlighting the need to understand the biological role of MeCP2 and particularly its involvement in the regulation of DNA methylation in the brain [72].

### DNA de-methylation

DNA de-methylation is a complex and not well-understood process. Recent evidence from studies on the adult mouse brain indicates that it is a multi-staged process, starting with the oxidation of 5-mC to form 5-hydroxymethylcytosine (5-hmC) [73]. Although 5-hmC can be formed during the process of active demethylation, it also acts as an important epigenetic mark, that is functionally different from 5-mC [74]. Interestingly, 5-hmC is prominent in the brain and plays an important role in neurogenesis [75]. The oxidation of 5-mC is executed by ten-eleven translocation (TET) enzymes, which, after the formation of 5-hmC, continue the oxidation process to subsequently form 5-formylcytosine (5-fC) and 5-carboxylcytosine (5-caC) [76]. However, a functional role for 5-fC and 5-caC as independent epigenetic markers has still to be elucidated [77]. In addition to the oxidation of 5-mC and 5-hmC, these marks can be de-aminated by activation-induced cytidine deaminase (AICDA) or by an apolipoprotein B mRNA editing enzyme, catalytic polypeptide-like protein (APOBEC), resulting either in thymine (T) or 5-hydroxymethyluracil (5-hmU) bases [73]. Regardless of the pathway, the formation of 5-caC, T or 5-hmU induces a base-to-base mismatch (i.e. 5-CaC:G, T:G or 5-hmU:G, respectively), resulting in the removal of the faulty base by thymine or uracil glycosylases [73,78,79], or the direct conversion of 5-fC and 5-caC back to C through deformylation or decarboxylation, respectively [76]. In addition to the aforementioned effector enzymes, the growth arrest and DNA damage-inducible 45 (Gadd45) family of proteins plays a pivotal role in the DNA demethylation process [80-83]. Although they do not exhibit enzymatic activity themselves, these proteins bind and direct the enzymatic activity of other proteins, such as cytidine deaminases and thymine glycosylases, to specific gene promoters.

#### ***DNA de-methylation and adult NSCs***

Hydroxymethylated DNA immunoprecipitation (hMeDIP) followed by high-throughput sequencing has recently started to reveal the genome-wide distribution patterns of 5-hmC in many tissues and cells. Using this technique, recent reports have suggested a functional role of 5-hmC during neural differentiation [75,84,85]. Specifically, one of these studies revealed dynamic changes in DNA hydroxymethylation during neural differentiation and identified differentially hydroxymethylated regions between ESCs and NPCs [84]. Of interest, 5-hmC is found in most tissues and its levels seem to be highest in brain, and enriched in synaptic genes [86].

As described above, the Gadd45 family of proteins mediates DNA demethylation. This family of proteins responds to changes in the environment by releasing gene repression at specific genes through the promotion of DNA demethylation [16,54,87]. Gadd45b is important specifically for the sequential steps of activity-induced neurogenesis in the adult hippocampus. Gadd45b is expressed in mature neurons in the hippocampus and neuronal activity is an important factor in controlling the rate of neurogenesis [81]. Ma and colleagues [81] studied activity-induced neurogenesis in the hippocampus of adult transgenic mice lacking Gadd45b. The increase in NSC proliferation after electroconvulsive therapy (ECT) observed in the hippocampus of control mice was significant decreased in Gadd45b knockout mice. Moreover, deficits in dendritic growth were observed in Gadd45b knockout mice, indicating that Gadd45b is important for neuronal maturation [81]. Methylated DNA immunoprecipitation (MeDIP) analysis revealed that Gadd45b is necessary for demethylation at different genes encoding growth factors involved in neurogenesis, including FGF1 [81], which regulates self-renewal and proliferation of NSCs similar to FGF2 [54]. These results indicate that Gadd45b is an immediate early gene expressed in mature neurons upon neural activity that subsequently regulates growth factor expression through DNA demethylation. Secretion of these growth factors, FGF1 specifically, induces increased neurogenesis in the surrounding neurogenic niche [81]. Therefore, Gadd45b provides a link between environmental signals (neuronal activity) and epigenetic DNA modifications that regulate adult neural stem cells.

### Histone modifications

In many cases, gene expression also depends on DNA accessibility, which is a.o., determined by chromatin structural organization. Chromatin is build up of multiple single nucleosomes consisting of 147 DNA base pairs (bp) wrapped around a group of proteins, called histones. Single nucleosomes contain a total of eight proteins, two copies of each histone 2A (H2A), histone 2B (H2B), histone 3 (H3) and histone 4 (H4). The amino-acid residues (N-terminal tails) of these proteins, or histone tails, are susceptible to multiple post-transcriptional modifications that regulate their function. Reversible modifications at the histone tails are established by different mechanisms such as acetylation, phosphorylation, methylation, ubiquitination and isomerization. The histone modifications induced by methylation and acetylation have been studied extensively and may either activate or repress expression of genes involved in neurogenesis [16,51].

Acetylation and methylation of histone tails is regulated by different enzymes. Histone acetyltransferases (HATs) and histone de-acetylases (HDACs) regulate acetylation levels while histone methyltransferases (HMTs) and histone demethylases (HDMs) regulate methylation. These enzymes target chromatin loci through specific associations with proteins that bind to target DNA sequences. Histone acetylation and methylation at certain loci may result in gene activation or repression. Histone “marks” associated with gene activation include acetylation of lysine 9 and 14 at H3 or tri-methylation of lysine 4 at the same protein. In contrast, di- or tri-methylation of lysine 9 or 27 at H3 is associated with repression of gene expression. These histone modifications induce alterations of the structural configuration of the nucleosome and change the accessibility of other transcriptional regulators to DNA. Together, the mechanisms of histone acetylation, de-acetylation, methylation and demethylation fine-tune gene expression and can regulate different stages of adult neurogenesis [16,51,52].

#### ***Histone acetylation and adult NSCs***

Acetylation of histone proteins is a dynamic process and especially the removal of acetylation marks by HDACs is important in neurogenesis [88]. Transcriptional repression through HDAC activity is essential for adult NSC proliferation and self-renewal. For example, the orphan nuclear receptor homologue of the Drosophila tailless gene (Tlx or NR2E1) regulates NSC self-renewal and interacts with different HDAC enzymes to regulate gene expression. Sun and colleagues [89] used ChIP analysis to show a direct interaction between Tlx and HDAC3, HDAC5 and HDAC7. These proteins are co-expressed in cultured adult mouse NSCs, and their expression is reduced upon NSC differentiation. Furthermore, these authors found that the cell-cycle regulator p21 was up-regulated in Tlx knockout mice and ChIP analysis revealed a common Tlx, HDAC3 and HDAC5 binding site in the p21 gene promoter. Moreover, treatment of cultured NSCs with the HDAC inhibitor valproic acid (VPA) induces p21 expression and increased acetylation of H4 at the p21 promoter [89]. Thus, both de-acetylation at the p21 promoter and activation of Tlx are necessary for inhibition of p21 expression. *In vitro* treatment of adult NSCs with VPA significantly reduced the amount of BrdU labeled cells, indicating a decrease in cell proliferation. Interestingly, both small-interfering RNA (siRNA) targeting Tlx and HDACs had the same effect [89]. Thus, the interaction of Tlx with HDAC3, HDAC5 and HDAC7 seems to be important for the regulation of genes involved in adult NSC proliferation.

A role for histone deacetylation in isolated adult SVZ NSCs is further supported by interesting observations done after treating these cells with the HDAC inhibitors sodium butyrate (NaB) and suberoylanilide hydroxamic acid (SAHA) [90]. Under these conditions, the authors observed impaired proliferation that was accompanied by a profound down-regulation of factors involved in stem cell maintenance and up-regulation of pro-neural factors. For example, the expression of Sox2 and the Notch effector transcription factors Hes1 and Hes5, involved in stem cell maintenance and proliferation, were down-regulated. Under induced differentiation conditions, SVZ NSCs pretreated with the HDAC inhibitor SAHA showed decreased glial and oligodendroglial differentiation compared to non-treated cells while neuronal differentiation was not affected [90]. These results support the role of HDAC activity in SVZ NSC proliferation, as was shown before by Sun and colleagues [89] and provide evidence for an additional role in adult NSC differentiation.

Increased neuronal differentiation at the expense of glial and oligodendroglial differentiation has also been observed in adult hippocampal NSCs treated *in vitro* with VPA that increased H3 acetylation levels and resulted in increased neuronal differentiation, even when factors favoring non-neuronal cell lineage differentiation were present [91]. Indeed, profound differences were observed when H3 and H4 acetylation levels were compared between NSCs and their progeny. Initially high H3 and H4 acetylation levels were found in undifferentiated NSCs and these levels remained relatively high in cells upon their differentiation into neurons. Lower levels of H3 and H4 acetylation were observed in cells differentiating into astrocytes or oligodendrocytes, suggesting that HDAC activity is crucial for NSC fate decisions. Thus, maintenance of histone acetylation seems important for neuronal lineage progression of adult NSCs, while histone de-acetylation appears important for astrocytic and glial lineage progression.

*In vivo*, BrdU analysis of the DG of VPA-treated adult rats showed a marked reduction in proliferation, accompanied by a significant increase in BrdU-labeled newborn neurons. Although astrocytic differentiation was unchanged, these results confirmed to a certain extent previous *in vitro* observations [91]. Similarly, Sun and colleagues [89] showed e.g. that HDAC expression, and thus probably histone acetylation, is decreased after neuronal differentiation of NSCs indicating an important role for histone acetylation in the regulation of NSC differentiation. Additional *in vitro* evidence supporting this notion was obtained using isolated NSCs from the adult SVZ [92]. In these experiments, treatment of NSC for the SVZ cells with SAHA increased their neuronal differentiation B [92].

HDAC2 is specifically important for neuronal maturation in both the adult SGZ and SVZ. HDAC2 is highly expressed in dividing cells within these areas. Low HDAC2 expression is associated with NSC quiescence, while higher expression levels are found in transit amplifying cells and HDAC2 remains present upon differentiation [93]. Deletion of HDAC2 in mice reduces total HDAC activity in the OB and hippocampal areas accompanied by a significant reduction in newborn neuron numbers and increases in cell death. In contrast, there was a significant increase in the proliferation rate of transit amplifying cells, as determined by the amount of cells in the S-phase of the cell cycle. This increased proliferation but defective neuronal generation in HDAC2 deficient mice is thought to result from the lack of gene repression by HDACs. The transcription factor Sox2 is expressed in wild type NSCs and its expression decreases upon progression to neuroblasts. However, in HDAC2 deficient mice, Sox2 expression was observed in neuroblasts present in the DG. This observation indicates that insufficient histone deacetylation of genes that are usually repressed by HDAC2 in cells differentiating towards the neuronal fate, like Sox2, may impair their maturation but increase their proliferation capacity. Importantly, although deletion of HDAC2 impaired neuronal maturation in the adult brain, deletion of HDAC2 did not change neurogenesis during embryonic development. Therefore, the requirement for HDAC2-dependent regulation of proliferation-related genes, allowing proper neuronal differentiation, seems specific for adult neurogenesis [93]. Thus, although several epigenetic mechanisms regulating embryonic neurogenesis are conserved into adulthood, also new mechanisms seem to emerge that regulate adult NSCs specifically.

The activity of several HATs has been studied *in vivo* as well [94]. The Querkopf (Qkf) protein is a member of the MYST family of HATs and it is a transcriptional activator with histone acetylase activity. During embryonic development, Qkf is expressed throughout the brain but its expression is restricted to neurogenic areas in the adult brain. In the SVZ of the adult brain Qkf is expressed in type A, B and C NSCs. A 90% reduction in Qkf transcription is observed in mice carrying hypomorphic Qkf alleles. This reduction is associated with decreased NSC proliferation and alterations in the proportions of the cell types derived from them, suggesting that defective neurogenesis in the OB of adult Qkf-deficient mice may result from a decrease in the proliferative NSC population and alterations in the cell progeny derived from it [94]. In addition, isolation of SVZ NSCs from Qkf-deficient mice showed impaired neuronal differentiation in vitro, while Qkf overexpression increased neuronal differentiation [94]. This indicates that the level of Qkf, and presumably of Qkf-mediated histone acetylation, regulates neuronal differentiation of adult NSCs in the SVZ. A similar impairment in neuronal differentiation was observed in isolated cells *in vitro*[94]. In conclusion, these results suggest a role for histone acetylation in neuronal differentiation, in line with previous studies where increased acetylation induced by HDAC inhibition increases neuronal differentiation.

#### ***Histone methylation and adult NSCs***

Adult neurogenesis is under tight epigenetic control of histone methylation that is regulated by two antagonistic complexes. The Polycomb-group (PcG) protein complex, that promotes histone 3 lysine 27 tri-methylation (H3K27me3), and the Trithorax-group (TrxG) protein complex, that promotes histone 3 lysine 4 tri-methylation (H3K4me3). Both are part of an evolutionarily conserved chromatin remodeling system that silences or activates gene expression, respectively. Together, these histone methylation events regulate the establishment and maintenance of different cell states in NSCs [51,54,95].

The PcG member B lymphoma Mo-MLV insertion region 1 homolog (Bmi-1) is required for postnatal NSC self-renewal. *In vitro*, Bmi-1 overexpression in NSCs isolated from the adult mouse SVZ increases neurosphere formation and self-renewing capacity of these cells [96]. Moreover, when differentiation was induced after five culture passages, the differentiation capacity of wild type NSCs was very low, while Bmi-1 overexpressing NSCs produced both glia and neurons under the same experimental conditions. Both immature and mature neuronal markers were expressed in these cultures. *In vivo* overexpression of Bmi-1 showed a similar increase in NSC proliferation in the SVZ and RMS [96]. This indicates that increased H3K27me3 induced by Bmi-1 overexpression could affect the expression of genes important for NSC proliferation and differentiation both *in vitro* and *in vivo*. In support, proliferation within the SVZ is decreased in adult Bmi-1 deficient mice [97]. In addition, NSCs isolated from Bmi-1 deficient mice showed decreased proliferation and self-renewal capacity *in vitro*, compared to wild type cells [97]. Although direct histone methylation measurements were lacking in this study, Bmi-1 is part of the PcG complex that catalyzes H3K27 tri-methylation, indicating that impairment of repressive histone methylation due to loss of Bmi-1 may be responsible for the results observed. Interestingly, Bmi-1 deficiency has been associated with increased expression of cell-cycle inhibitors such as p16 (Ink4a) and p19 (Arf), and accurate repression of these genes by Bmi-1 represents a critical mechanism by which Bmi-1 drives NSC self-renewal [98].

Recent observations have shown that the TrxG member mixed-lineage leukemia 1 (Mll1) is required for adequate neurogenesis progression [99]. Mll1-deficient NSCs purified from the SVZ survived, proliferated and efficiently differentiated into glial lineages but their neuronal differentiation was impaired. In Mll1-deficient cells, the expression of the early proneural Mash1 and gliogenic Olig2 expression was preserved, but Dlx2, a key downstream regulator of SVZ neurogenesis, was not detected. In line with these observations, neurogenesis could be rescued by Dlx2 overexpression, demonstrating the crucial role of Mll1 in controlling Dlx2 expression and thus progression towards a neuronal phenotype. Indeed, ChIP analysis showed direct interactions of Mll1 with the Dlx2 gene promoter and Dlx2-regulatory sequences were bivalently marked by both H3K4me3 and H3K27me3 in Mll1-deficient cells. This bivalent histone methylation pattern resulted in the Dlx2 gene failing to properly activate, demonstrating the relevance of epigenetic regulation of Dlx2 in controlling adult neurogenesis in the SVZ [99]. *In vivo*, Mll1 deficiency decreases the size of neurogenic regions in the postnatal brain including neuronal number, with a sharp decrease in the amount of newly formed neurons in the OB. However, in the SVZ, DCX positive cells are increased in number, indicative of an impaired migratory capacity. Moreover, continuous expression of transit-amplifying cell characteristics in these DCX expressing neuroblasts suggests that gene repression upon differentiation was impaired, which may provide a plausible explanation for the impaired differentiation and migration observed in Mll1 deficient neuroblasts [99]. Thus, Mll1 expression and histone methylation catalyzed by the TrxG complex seems to be an important regulator of postnatal neurogenesis in the mouse SVZ.

Wu and colleagues [62] have demonstrated the ability of DNMT3a to interact with histone methylation. Whereas DNA methylation at promoter regions generally prevents the binding of transcription factors and inhibits gene expression, Wu and colleagues [62] showed that DNMT3a activity correlates with increased expression of neurogenic genes. The increased expression of these neurogenic genes seems to be mediated through an interaction between DNA methylation and histone methylation. ChIP analysis showed that loss of DNMT3a increased binding of the PcG complex Polycomb repression complex 2 (PRC2) to DNMT3a targets, which was accompanied by increased H3K27me3 levels and decreased target gene expression. This effect was specific for DNMT3a targets since binding of PCR2 and H3K27me3 levels did not change at non-DNMT3a targets. In support of this conclusion, restoration of DNMT3a activity functional rescued by introduction of wild type DNMT3a reversed the abnormally increased H3K27me3 levels and PRC2 occupancy at down-regulated DNMT3a target genes in the DNMT3a knock-out NSCs. These results indicate that methylation by DNMT3a may antagonize the repression of gene activity mediated by PcG complex binding and H3K27me3 establishment in NSCs [62] and support a function for DNMT3a in the repression of genes regulating NSC self-renewal and activation of neurogenic genes, thereby regulating neuronal differentiation.

### MicroRNAs

A third epigenetic mechanism capable of controlling the neurogenic process involves non-coding RNAs. Transcription of non-coding DNA regions generates several classes of non-coding RNAs. Small non-coding RNAs, such as siRNAs, small nucleolar RNAs (snoRNAs), piwi-interacting RNAs (piRNAs), small modulatory RNAs (smRNAs), repeat-associated small interfering RNAs (rasiRNAs), transcription initiation RNAs (tiRNAs), small double-stranded RNA (dsRNAs) and microRNAs (miRNAs) are all expressed in the brain [100]. More specifically, the 21–25 nt long miRNAs have been linked to the regulation of gene expression during adult neurogenesis, acting post-transcriptionally, usually through their binding to the 3′ un-translated regions (3′ UTR) of their target mRNAs. In most cases, the binding of a miRNA to an imperfect complementary gene transcript results in the repression of translation of the target mRNA. Since most miRNAs form imperfect base-pairs with their mRNA targets, a single miRNA is capable of regulating a large number of different genes. About 2019 unique human miRNAs and 1265 mature miRNAs in mice have been identified to date [101].

Modulation of gene expression of different signaling molecules involved in the neurogenic process, as well as of other epigenetic mechanisms present in the brain, implicate an important function of miRNAs in adult neurogenesis. Since a number of excellent reviews [51-54,95,100] have highlighted the roles of miRNAs in the regulation of gene expression in NSCs, we present in the next section only a short overview of the published data.

#### ***MicroRNAs and adult NSCs***

Functional studies of different miRNAs demonstrate their importance for different stages of adult neurogenesis. Let-7b, miR-9, miR-106b, miR-137, miR-184 e.g., are involved in the proliferation of adult mouse NSCs. An additional role for miR-9, miR-34a, miR-137 and miR-184 as well as for miR-124 have been found in neuronal differentiation. Moreover, miR-137 is involved in synaptogenesis and miR-132 regulates both synaptogenesis and neuronal network integration of adult mouse NSCs [100], while miR-34a and miR-125b modulate dendritogenesis and spine morphology [102]. We here focus on well-studied miRNAs with a key role in adult neurogenesis, e.g. miR-34a, that was recently implicated in aging and neurodegeneration in Drosophila, is an essential miRNA, particularly in the developing brain [103].

MiR-34a regulates neuronal differentiation via Notch signalling by repressing the γ-secretase inhibitor numb homolog (Drosophila)-like (*NUMBL*) [104]. Overexpression of miR-34a increases neurite elongation of mouse NSCs [105]. MiR-34a modulates the expression of synaptic targets including synaptotagmin-1 and syntaxin-1A while its target SIRT1 may mediate the effects on neurite elongation. Overexpression of miR-34a further alters hippocampal spinal morphology, and subsequent electrophysiological function of dendritic spines [106].

MiR-125b is another brain-enriched miRNA, abundantly expressed in the foetal hippocampus under physiological circumstances [107-109]. MiR-125b levels increase during *in vitro* differentiation of embryonic stem cells [110]. Moreover, miR-125b is downregulated in cerebellar neuronal progenitors, increasing with differentiation, thereby allowing cell maturation and growth inhibition [111]. MiR-125b functions by suppressing Nestin expression, thus modulating proliferation and differentiation of neural stem and progenitor cells, as well as migration of the cell types derived from them [112]. Furthermore, the regulatory function of miR-125b on dendritogenesis could be partly attributed to the fact that a subset of its repressed targets, such as itchy E3 ubiquitin protein ligase (ITCH) and diacylglycerol O-acyltransferase 1 (DGAT1), in turn antagonize neuronal genes in several neurogenic pathways. Therefore, their translational repression by miR-125b suggests a positive role for miR-125b in neurite outgrowth and differentiation [113].

MiR-132 is a brain-enriched miRNA centrally involved in the regulation of neuronal plasticity upon neuronal activation [114]. Overexpression of miR-132 in cultured hippocampal neurons demonstrates that miR-132 modulates short-term synaptic plasticity [115], while overexpression *in vivo* triggers an increase in dendritic spine density [116]. MiR-132 has been proposed to differentiate neuronal stem cells specifically into dopaminergic neurons via a direct posttranscriptional repression of the nuclear receptor subfamily 4, group A, member 2 (NR4A2, also known as Nurr1) [117]. MiR-132 is also required for normal dendritic maturation in newborn neurons in the adult hippocampus and indirectly participates in CREB-mediated signaling [118]. More specifically, CREB-induced transcription of miR-132 results in a decrease of MeCP2 expression and a subsequent decrease in brain-derived neurotrophic factor (BDNF) due to de-repression of REST [119]. On the other hand, miR-132 expression is greatly enhanced via the ERK1/2 pathway by neurotrophins, such as BDNF, thus forming a negative regulatory feedback loop [120].

Although MiR-124 is abundantly expressed in the adult brain, its expression in different isolated cell types of the adult mouse SVZ indicates an important role in neuronal differentiation. While expression was absent in both type B and C cells, miR-124 expression was observed at the transition from type C transit amplifying cells to type A neuroblast cells. Upon further differentiation, expression increases [121]. Separation of the neuroblast population based on their cell cycle stage indicated by a DNA dye shows increasing miR-124 levels from S/G2-M phase to G0/G1 phase. Thus, miR-124 expression increases at the transition from type C to type A cells and furthermore increases upon cell cycle exit of the neuroblasts. An *in vitro* knock-down of miR-124 decreases the amount of neuroblasts exiting the cell cycle, while the amount of proliferating type C and A cells increases. This indicates that miR-124 expression is specifically important for the transition from proliferating neuroblasts to differentiated neuroblasts that have left the cell cycle. Computational analysis of miR-124 targets identified the Sox9 transcription factor, that is involved in NSC self-renewal, the Notch-ligand Jagged-1 and the transcription factor Dlx2. MiR-124 targeting of Sox9 was studied in more detail [121]. While differentiating NSCs expressing miR-124 still express Sox9 mRNA, Sox9 protein expression is repressed. This observation supports post-transcriptional repression of Sox9 by miR-124 at the transition from proliferating to differentiating neuroblast cells.

Similarly, repression of mRNA translation by miR-9 is important for neuronal differentiation. Expression of this miRNA has been observed in the neurogenic regions of the brain [122]. Although different miR-9 targets have been identified to regulate this process, miR-9 expression, like miR-124, increases upon neuronal differentiation. Functional analysis of miR-9 in isolated adult mice forebrain NSCs supports its role in neuronal differentiation. While miR-9 overexpression reduced NSC proliferation and increased neuronal differentiation, miR-9 knock-down showed opposite effects. MiR-9 overexpression was accompanied by a reduction in expression of the Tlx receptor that is involved in NSC maintenance, as discussed before. ChIP analysis showed that miR-9 targets Tlx at its 3′UTR, inducing translational inhibition. MiR-9 thus negatively regulates Tlx expression and reduces NSC proliferation but increases neuronal differentiation [122]. Another miRNA targeting Tlx is Let-7b. Increased expression has been observed upon neuronal differentiation similar to miR-124 and miR-9. A knock-down of Let-7b enhances NSC proliferation and decreases neuronal differentiation, while again overexpression shows the exact opposite [123].

Additional functions resulting from the combined actions of miR-9 and miR-124 in neuronal fate progression were demonstrated in a reprogramming study of isolated human fibroblasts [124]. Here the authors showed that miR-9 and miR-124 are capable of inducing a neuronal fate conversion. Combined expression of these miRNAs with transcription factors important for neurogenesis enhanced the rate of conversion of these cells into the neuronal lineage, which was accompanied by an increased maturation of the differentiated neurons. Strikingly, neurogenic transcription factor expression alone did not induce the conversion of these fibroblast cells into the neuronal fate [124]. Thus, the combination of miR activity regulating gene translation and the regulation of gene expression by different transcription factors act together to induce a neuronal fate conversion. This study emphasizes the importance of these miRNAs in the induction of neuronal fate.

Other miRNAs regulate different stages of adult neurogenesis acting on different targets during the process of neuronal maturation [125]. Adult mice hippocampal NSCs were isolated and used to identify lineage specific miRNAs. To this aim, miRNA expression patterns of differentiated astrocytes and neurons were compared by qPCR and miRNAs specifically enriched in the neuronal lineage were further investigated. Following this approach, miR-137, specifically enriched in neurons, was implicated in neuronal maturation. *In vivo* overexpression of this miRNA in newborn neurons of the adult mouse DG decreased their dendritic complexity, dendritic spines and length of the maturated neurons. This indicates that the maturation process in the miR-137 overexpressing cells was impaired. The increase in miR-137 seems to disrupt the sequential events of neuronal maturation leading to structural alterations. *In vitro* analysis of miR-137 expression confirmed enrichment in dendrites of differentiated neurons, indicating a role in development of these dendrites as was observed *in vivo *[125]. Underscoring its importance in neurogenesis, miR-137 targets the mind bomb 1 (MIB1) protein, an ubiquitin ligase essential in neurodevelopment [125] and miR-137 post-transcriptionally represses the expression of Ezh2, a histone methyltransferase and Polycomb group protein, resulting in a global decrease in histone H3K27me3. Furthermore, miR-137 is epigenetically regulated by MeCP2, a DNA methyl-CpG-binding protein, a mechanism we discussed before and in the next section [126]. Although in-depth mechanistic studies of miRNA functioning will have to be done in order to understand the complete regulation network, overall, the studies discussed in this section suggest that miRNAs are capable of regulating NSCs at different stages. Subsequent identification of miRNA targets might contribute to unravel the control of neurogenesis at the molecular level.

#### ***Epigenetic interplay in the regulation of adult NSCs***

In addition to gene expression regulation, miRNAs also interact with, and regulate epigenetic mechanisms such as DNA methylation and histone modifications, with possible consequences for AD [127]. These interactions are considered central to understanding the regulation of gene-expression networks during neurogenesis. For instance, two epigenetic regulators that have been found to interact are MBD1 and miR-184. MBD1 knock-out *in vivo* and acute knock-down of MBD1 *in vitro* induce significant increases in miR-184 expression [128]. In contrast, *in vitro* overexpression of MBD1 decreases miR-184 expression. Indeed, the genomic region surrounding the miR-184 gene contains high CpG-rich areas and ChIP analysis of wild type NSCs showed MBD1 binding surrounding the miR-184 genomic area. The increase in miR-184 expression observed in MBD1 deficient NSCs was accompanied by increased H3K4me3 and H3K9Ac and decreased H3K27me3 surrounding the miR-184 genomic region [128]. These results indicate that MBD1 may regulate miR-184 expression by interacting with histone modification mechanisms. MBD1 seems to antagonize H3K4me3 and thereby inhibit miR-184 expression using a mechanism different from the DNMT3a-mediated antagonism of H3K27me3 discussed in previous sections [62]. Overexpression of miR-184 showed repression of astroglial and neuronal lineage genes and decreased differentiation of adult NSCs *in vitro*. Moreover, NSC proliferation and neurosphere formation were increased. *In vivo*, BrdU analysis after miR-184 overexpression in the DG indicated an increase in NSC proliferation while the percentage of differentiating cells was decreased [128]. As Zhao and colleagues [64] and Singh and colleagues [61] showed before, MBD1 regulates neuronal differentiation. These results suggest that the regulation of neuronal differentiation mediated by miR-184 may involve its regulation by MBD1 and modifications of histone marks.

Thus, the interplay between MBD1, miR-184 and histone modification mechanisms seem to maintain, at least in part, the balance between NSC proliferation and differentiation. Moreover, as discussed before, MBD1 targets FGF2, important for NSC proliferation [65]. Repression of this growth factor by MBD1 is necessary for proper neuronal differentiation, adding an additional player within this molecular network regulating neuronal differentiation of adult NSCs. In support of this hypothesis, activity dependent DNA demethylation by Gadd45b increases expression of a growth factor similar to FGF2 [81]. Based on the previous discussion, these complex interactions between epigenetic mechanisms could explain, at least in part, the release of repression on proliferation/differentiation genes through histone modifications and decreased MBD1 binding due to DNA demethylation.

## Alzheimer’s disease

AD is a neurodegenerative disorder characterized by severe and progressive memory deficits, accompanied by atrophy of specific brain regions and extensive neuropathology and gliosis. It is initially identified by impaired episodic memory that worsens with the accumulative neurodegeneration [129]. The disease is characterized by the accumulation of β-amyloid (Aβ), a peptide derived from the proteolysis of the amyloid precursor protein (APP), which forms the main components of extracellular senile plaques, and the accumulation of intracellular neurofibrillary tangles (NFTs), which mainly consist of microtubule-associated protein tau, that is hyperphosphorylated and organized in paired helical filaments [130].

### AD and neurogenesis

Several studies have shown that, in addition to age, neuroinflammatory and neurodegenerative processes have a pronounced influence on adult neurogenesis [131]. In AD, biochemical and histological approaches have provided contradictory results when comparing animal models and studies on human brain. Studies in AD were not only hampered by obtaining standardized human tissue of sufficient quality, but also by the lack of reliable makers to identify the different stages of the neurogenic process in post-mortem tissue. Although specialized markers from the tumor field have been promising, methodological issues of post-mortem delay, specificity and fixation are not trivial and so far, only a few studies have reported changes in proliferation or young neuronal markers in AD brain. One report showed increases in various immature neuronal markers in senile AD, suggesting that neurogenesis could be increased in late AD [132]. In a study in younger, presenile patients, these results could not be replicated [133]; although a significant increase in the number of Ki-67+, proliferating cells was found, these cells were mostly associated with glia and the vasculature (Marlatt et al., submitted 2014). Later studies have used markers like Musashi-1, nestin and PSA-NCAM to show that neurogenic abnormalities in AD differ between phases and areas of neurogenesis and stages of AD: while hippocampal stem cells (Musashi-1) decrease, proliferation increases and differentiation/migration phase as well as axonal/dendritic targeting (DCX and β-III-tubulin) remain unchanged, suggesting an attenuation of stem cells together with compensatory increases in proliferation that, however, does not result in increases in differentiated new neurons in AD [134]. Similar findings exist on microtubule-associated protein isoforms some of which represent immature neuronal markers, like the MAP2c isoform. Another study reported a decrease in DCX- and sex determining region Y-box 2 (Sox2)-positive cells in human AD but an increase in bone morphogenetic protein 6 (BMP6) levels that was also found in APP transgenic mice, suggesting a role in defective neurogenesis in AD [135]. Collectively, these findings suggest that proliferating cells in the AD dentate gyrus do not become mature neurons [136-138]. Also, it is yet clear whether this represents a compensatory mechanism in response to neurodegeneration or an effect induced by the medication the patients received before death.

On the other hand, a decline in proliferation in the SVZ has also been shown in AD [139,140]. More recently, Perry and colleagues [134] observed that while hippocampal stem cells decrease, proliferation increases and differentiation/migration phase as well as axonal/dendritic targeting remain virtually unchanged, suggesting a decrease in NSC numbers accompanied by compensatory increased proliferation which again, may not result in increases in migratory neuroblasts and/or differentiated neurons in AD. Additionally, they suggested that neurogenic abnormalities in AD would differ between phases and areas of neurogenesis and stages of AD.

Transgenic mice expressing human APP and presenilin-1 (PS1) genes with familial AD associated mutations, exhibit early and progressive accumulation of Aβ, possibly resulting in compromised neocortical synaptic plasticity and synaptic dysfunction, traits similar to those observed in AD patients [141]. In most of these experimental models decreased proliferation, differentiation and survival have been described but this depends on the stage of the disease and the extent of pathology [21]. Moreover, decreased neurogenesis and increased astrogenesis were found in APOE knock-in mice [142]. Interestingly, mice solely expressing human mutated PS1 presented with an age-related increase in hippocampal granule cell numbers, suggesting a beneficial role for the PS1 gene on neurogenesis [143]. In another study using PS1/PS-2 double-knockout mice, robust AD like pathology is found [144], notably in absence of beta-amyloid deposition, and the authors observed enhanced neurogenesis in the early stages of neurodegeneration. However, this increase could not be detected anymore at later disease stages, possibly due to a decreased survival of the newly generated neurons [144]. Because these dynamic changes in neurogenesis were correlated with the severity of neuronal loss in the DG, the authors concluded that neurogenesis may work as a self-repairing mechanism to compensate for neurodegeneration.

Interestingly, a recent study designed to study the effect of different variants of hAPP on morphological and functional parameters during GC development found that some hAPP cleavage products, such as the β-C terminal fragment (β-CTF) C99, induced a substantial reduction in glutamatergic connectivity in 21 day-old newborn neurons in the SGZ [145], a period of active dendritic growth and synaptogenesis [146]. Importantly, the strength of glutamatergic inputs recovered in mature, 35-day-old neurons and the delay in glutamatergic synaptogenesis observed by the authors was paralleled by a decrease in dendritic length with no changes in spine density, suggesting that hAPP may be able to affect dendritic development under certain circumstances [145].

All in all, the experimental evidence discussed in this section suggests that a better comprehension of the signaling mechanisms that modulate neurodegeneration and neurogenesis in AD could provide us with new candidate targets for future studies on AD neurobiology and treatment.

### AD and epigenetics

The etiology and pathophysiology of AD, including aberrant processing of amyloid and tau, are not well understood. Recently, some studies have pointed out that epigenetic changes could be involved in these processes that will be discussed below.

#### ***AD and DNA methylation***

Initial epigenetic investigations pertaining to AD focused on DNA methylation of the APP gene and illustrate the complexity and difficulty of investigating the epigenetics of the multifactorial and heterogeneous affliction that is AD. West and colleagues [147] observed hypomethylation of the APP gene promoter in an AD patient, whereas Barrachina and colleagues [148] did not find any significant AD-related abnormalities in methylation of the APP promoter region. They also did not find any abnormal methylation patterns in the MAPT and PS1 genes, even when looking at different stages of the disease. While this group did report the presence of high and low methylated CpG sites in and around the APP promoter region, Brohede and colleagues [149] found no methylation at all at the investigated CpG site in the APP gene. Interestingly, Tohgi and colleagues [150] have found an age-related decrease in cytosine methylation in the promoter region of the amyloid precursor protein (APP) gene in the human cerebral cortex. Additionally, they observed abnormal cytosine methylation in the promoter region of the tau gene in the aged human cerebral cortex [151].

Although it remains to be elucidated whether the APP gene is specifically regulated by DNA methylation or not, strong evidence suggests that DNA methylation is disrupted in AD. Pioneering studies have shown that S-adenosylmethionine (SAM), a methyl donor crucial for DNMTs activity, is severely reduced in AD [152]. Later, the relation of this finding with actual DNA methylation was corroborated by the detection of decreased global DNA methylation in the AD brain [153,154]. Additional studies have specifically investigated the hippocampus, one of the brain regions strongly affected by AD and found increased levels of 5-mC [155] and DNMT3a [63] in the hippocampus of aging mice, but reduced 5-mC levels in APP/PS1 transgenic mice (Chouliaras et al., submitted, 2014) and in the hippocampus, entorhinal cortex and cerebellum of AD patients [156,157]. Furthermore, DNA methylation in AD seems to particularly involve DNMT3a, as the presence of a tagSNP in the DNMT3a gene correlated with cognitive decline in MCI patients (Chouliaras et al., submitted 2014).

Remarkably, Aβ itself has been shown to affect DNA methylation [158]. Aβ seems to induce global DNA hypomethylation, while its effect on specific genes is more complex. Indeed, the NEP gene seems to be hypermethylated under influence of Aβ, repressing its transcription [158]. This interaction between Aβ and NEP might be of crucial importance for AD pathology, as the NEP gene encodes for neprilysin, one of the primary enzymes involved in Aβ degradation.

Although the consequences of aberrant DNA methylation associated with AD remain to be fully elucidated, some affected genes have been identified. Siegmund and colleagues [159] found SORBS3 to be hypermethylated, while S100A2 was hypomethylated, possibly reflecting an acceleration of the age-related changes in the normal brain. SORBS3 encodes a cell adhesion molecule and decrements in its expression seem to contribute to the synaptic abnormalities associated with AD [160]. Increased expression of S100A2, which encodes a calcium binding protein, is associated with corpora amylacea formation [161]. In addition, Scarpa and colleagues [162] showed that PS1 was hypomethylated. As the protein encoded by PS1 is part of the enzymatic complex responsible for Aβ production, increased PS1 expression may enhance Aβ formation. Of note, one study comparing human postmortem frontal cortex genome-wide DNA methylation profiles between late-onset AD and 12 cognitively normal controls found widespread, albeit modest, discordant DNA methylation independent from DNA methylation changes with age [163].

#### ***AD and DNA hydroxymethylation***

DNA hydroxymethylation is not as well studied as DNA methylation, and neither in relation to AD. Nevertheless, studies of DNA hydroxymethylation in the hippocampus suggest a pattern similar to DNA methylation: increasing levels with normal aging [155,164,165], but strongly decreased levels in APP/PS1 mice (Chouliaras et al., submitted 2014) and AD patients [156,157]. Interestingly, Münzel and colleagues showed that levels of 5-hmC increase with age [164]. The importance of DNA hydroxymethylation in AD is further stressed by the discovery of a single nucleotide polymorphism (SNP) in the TET1 gene, which protein catalyzes the conversion of 5-mC into 5-hmC, associated with late onset AD [86,166]. While the functional impact of changes in DNA hydroxymethylation associated with AD largely remain to be explored, the findings discussed in this section further support the notion of a widespread failure of the epigenetic regulatory system in AD.

#### ***AD and histone modifications***

Besides DNA methylation, a growing body of evidence suggests that alterations in histone acetylation are among the basic molecular mechanisms underlying AD pathogenesis. Histone acetylation is significantly lower in the temporal lobe of AD patients compared to aged controls [167]. Furthermore, Marques and colleagues [168] showed that increased levels of beta-secretase 1 (BACE1), a protease that cleaves APP in the amyloidogenic pathway, are seen in peripheral blood mononuclear cells of AD patients and increased BACE1 promoter accessibility are associated with increased histone H3 acetylation. These findings are supported by other observations showing aberrant histone acetylation levels in animal models of AD [169]. Interestingly, there is some evidence that dysregulation of histone H4 lysine 12 (H4K12) acetylation is implicated in learning impairment in aged mice. Peleg and colleagues [170] observed that differential gene expression and abnormal H4 acetylation were associated with impaired memory function in contextual fear conditioning in aged mice. Interestingly, these deficits were counteracted by the application of HDAC inhibitors into the hippocampus [170]. Importantly, chronic systemic inhibition of HDAC reverts the cognitive deficit observed in APPswe/PS1dE9 transgenic mice in the contextual fear conditioning model [171]. Unfortunately, the identity of the specific HDAC(s) that is responsible for the memory impairments remains unknown because these studies have mostly used non-selective HDAC inhibitors.

More recent studies have indicated that HDAC2, crucially involved in the regulation of memory and synaptic plasticity, might be directly implicated [172]. Gräff and collaborators investigated the role of HDAC2 in AD [173]. Using CK-p25 mice as model for AD-like neurodegeneration, they found a significant increase of HDAC2 in the hippocampus and prefrontal cortex of these mice. In contrast, no significant changes in HDAC2 expression were detected in the amygdala, an area not affected by neurodegeneration in this animal model. When these authors investigated the functional impact HDAC2 dysregulation, they found that H2bK5, H3K14, H4K5 and H4K12 were all hypoacetylated in CK-p25 mice. Importantly, increased HDAC2 binding and hypoacetylation negatively correlated with activated RNA Polymerase II binding and mRNA expression in genes related to learning, memory and synaptic plasticity [173]. These observations were confirmed by HDAC2 knockdown, which successfully restored synaptic plasticity and cognitive performance in CK-p25 mice. In addition, Gräff and colleagues [173] investigated the effects of two neurotoxic stimuli associated with AD, hydrogen peroxide and Aβ, on HDAC2 expression in primary hippocampal neurons. They found that these noxious stimuli increased HDAC2 levels in cells, an event likely resulting from glucocorticoid receptor (NR3C1) activation in response to the neurotoxic stimuli, thus linking AD hallmarks to aberrant epigenetic regulation possibly mediated by NR3C1. Finally, Gräff and colleagues [173] validated their findings in postmortem human brain samples from sporadic AD cases at different Braak stages. These experiments revealed that HDAC2 levels are significantly increased in the hippocampus and entorhinal cortex, areas known to be affected in AD. Moreover, HDAC2 levels were elevated in all Braak stages, including I and II, indicating that deleterious HDAC2 activity might be one of the earlier events in the development of AD.

#### ***AD and microRNAs***

Apart from their involvement in regulating neurogenesis in normal conditions mentioned in previous sections, miRNAs have also been shown to be involved in AD pathogenesis. We and others have recently reviewed the experimental evidence supporting this conclusion [127], so we only discuss some relevant examples here. For instance, miR-15, miR-16, miR-132 and miR-497 have been associated with tau regulation, whereas miR-106a, miR-106b, miR-107, miR-124, miR-137, miR-153, miR-195 and miR-520c have been linked to APP metabolism and Aβ production [174]. More specifically, a role for miR-132 in the regulation of alternative splicing of tau exon 10 has been demonstrated by studying its repression of the polypyrimidine tract binding protein 2 (PTBP2) transcript. This repression interfered with the physiological phosphorylation of tau, thus linking aberrant miR-132 functioning to possible disease state [175]. In the same study, members of the miR-16 family (miR-16, miR-15, miR-195 and miR-497) were identified as regulators of ERK1 and therefore tau phosphorylation in neuronal cells *in vitro*, including primary rat neurons. An additional link between miR-16 expression and AD pathology was introduced by Liu and colleagues [176]. In this study, miR-16 overexpression reduced APP levels in the brains of senescence-accelerated mouse prone 8 (SAMP8) mice, another animal model of age-related behavioral deterioration and AD-associated neurodegeneration that displays deficits in learning and memory [177].

Regulation of Aβ production further implicates miRNA function in AD via different mechanisms. For instance, endogenous miR-106a, miR-153, and miR-520c downregulate APP levels in human neurons by directly targeting the 3′ UTR of the APP mRNA [178,179] and thus reducing Aβ levels. Suppression of BACE1 translation by miR-195 and miR-124 also reduces Aβ production [180,181], while miR-137 and miR-181c indirectly regulate Aβ production via modulation of serine palmitoyltransferase (SPT) levels [182]. Lastly, the expression of certain miRNAs is affected by the presence of Aβ. miR-106b expression appears to be induced in APPswe/PS1dE9 brains due to increased Aβ42 oligomers [183], whereas miR-9 and miR-181c are downregulated in cultured hippocampal neurons exposed to Aβ, providing another link to the pathogenesis of AD [184].

Interestingly, while some of the miRNAs implicated in AD are also involved in other neurodegenerative diseases, such as Mild Cognitive Impairment (MCI) or Parkinson’s Disease (PD), some seem to be more specific to AD itself. Recently, Leidinger and colleagues pinpointed a ‘12-miRNA signature’ in AD using next generation sequencing (NGS) to trace miRNAs from blood samples of 44 AD patients and 22 age-matched healthy controls [185]. The signature consisted of miRNAs that were differentially expressed strictly in AD, including miR-26a, -26b, -103a, -107, -112, -151a, -161, -532, -1285, -5010, let-7d and let-7f, thereby providing a tool to distinguish AD from other neurodegenerative diseases with a reasonable accuracy [185]. Of note, many of these 12 miRNAs may have distinct roles in neurodevelopmental pathways, such as neurite outgrowth, synaptic formation and neuronal migration, portraying the complex nature of AD and its implications in neuronal development.

### AD, Epigenetics and adult neurogenesis

Epigenetics and neurogenesis are areas of interest to AD, both from a pathophysiological as well as for a treatment perspective. These fields have, however, generally been investigated separately in relation to AD, despite the crucial role of epigenetic regulation in normal neurogenesis. As discussed above, DNA methylation is crucial for NSC fate determination, differentiation and migration, specifically implicating DNMT1 and DNMT3a [61,62]. However, how changes in their levels of expression or activity could be linked to AD pathogenesis or progression remains largely unknown. Interestingly, chronic stress, an environmental factor linked to an increased risk to develop AD [186], increases DNMT3a expression in the nucleus accumbens in rodents [187]. Moreover, the observation that brain SAM [152], 5-hmC and 5-mC levels [156] are drastically decreased in AD patients suggests that differentiation and migration of NSCs is impaired in end stage AD. Furthermore, decreased levels of DNA methylation in AD may interfere with MBD1 binding, which is important for newborn neuron survival and differentiation [64]. Indeed, most studies in mouse models of AD found decreased differentiation and survival of NSCs [21]. Various others, however, detected an AD-associated increase in proliferation, which could be considered a compensatory mechanism [132,134].

Investigations of the use of the HDAC inhibitor VPA as a potential treatment for AD have highlighted alterations in the intricate balance between proliferation and differentiation required for neurogenesis. While VPA seems to reduce NSC proliferation [89], it induces differentiation of neural progenitor cells, specifically enhancing the generation of new neurons, and suppresses progression towards the astrocyte and oligodendrocytes lineages [91]. Considering the detection of significantly decreased levels of histone acetylation in the temporal lobe of AD patients [167], it appears that histone acetylation may be impaired in AD. This impairment in histone acetylation hampers synaptic development in the hippocampus, which may in part explain the ability of VPA to improve memory deficits in animal models of AD [188].

Ogawa and colleagues [189] observed that neurons vulnerable to neurodegeneration in AD display signs of cell cycle activation, but fail to proliferate. They investigated this phenomenon and found that the phosphorylation of histone H3, a histone modification crucial for chromosome compaction during cell division, was increased, but appeared to be anomalously located in the neural cytoplasm. This ectopic localization of an epigenetic modification crucial for cell proliferation suggests that abnormal nuclear transport might play a role in the epigenetic regulation of neurogenesis in AD. In support of this hypothesis, Mastroeni and colleagues [190] recently found DNMT1 and RNA polymerase II to be abnormally sequestered in the cytoplasm in AD brains. Importantly, their observations points towards an Aβ-induced reduction in the expression of Ras-related Nuclear protein (RAN), a protein crucially involved in nucleocytoplasmic transport, as a major contribution to the apparently malfunctioning nucleocytoplasmic transport in AD.

## Future perspectives

There is no perfect animal model for sporadic AD to date, and those that exist mostly resemble rare familial variants of AD [191]. Nevertheless, animal models have and will certainly continue to play an important role in AD research [192,193]. Although sporadic AD is much less understood, recent evidence discussed in previous sections, suggests that epigenetic mechanisms may be involved in aspects of the etiology of AD [194]. Therefore, it might be fruitful to develop animal models of sporadic AD based on modulations of the cellular epigenetic machinery [195]. Such models could be achieved through the introduction of genetic mutations in genes encoding proteins or miRNAs that are involved in epigenetic regulation, pharmacologic induced dysregulation of the epigenetic machinery, or through RNA interference of components of the epigenetic apparatus. They may reflect the etiology of sporadic AD in the sense that they could include environmental factors, such as early or chronic stress.

A highly promising new addition to modeling techniques available for AD and an alternative to animal models are induced pluripotent stem cells (iPSCs). The procedure to produce stem cell-like cells from mouse fibroblasts was developed in 2006 by Takahashi and Yamanaka [196] and a year later they reported about their successful generation of human iPSCs [197]. This technique allows for the de-differentiation and reprogramming of somatic cells into iPSCs through the expression of a specific set of transcription factors (e.g. octamer-binding transcription factor 4 [OCT], SRY-related HMG-box gene 2 [SOX2], Krüppel-like factor 4 [KLF4] and cMYC) that induce the expression of pluripotency related genes and suppress lineage-associated genes. These iPSCs resemble embryonic stem cells (ESCs), in the sense that they can proliferate indefinitely and have the potential to differentiate into any type of cell [198]. Although this technique is still very time consuming and has a low throughput, it allows for a unique way of modeling elements of AD; through the generation of actual AD neurons from patient-derived iPSCs. Furthermore, this method allows to model sporadic AD, without the need of specific disease-inducing genetic mutations and the creation of transgenic animal models. However, in terms of models that resemble or mimic epigenetic mechanism linked to AD, it is worth noting that reprogramming of somatic cells into iPSCs implies a significant resetting of their epigenetic information [199].

Despite ongoing discussions about the exact nature of iPSCs, the best procedure to generate them, genetic stability, reproducibility of the resulting cell line and how well re-differentiated iPSCs resemble the target cells, some interesting discoveries have been done with AD patient-derived iPSCs [200]. One study, using iPSC-derived purified neurons from familial (caused by a duplication of the APP gene) and sporadic AD patients and non-demented controls, found that especially for familial AD, and to a lesser extend sporadic AD neurons displayed higher amounts of Aβ, phospho-tau and active glycogen synthase kinase-3β (GSK-3β), all pathological markers of AD [201]. Remarkably, GSK-3β activity controls the expression of O(6)-methylguanine DNA methyltransferase (MGMT) a methyltransferase which repairs DNA damage specific to O(6)-position of guanine [202,203]. Another study induced a neuronal phenotype in human isolated fibroblasts from familial AD patients (with PS1 or PS2 mutations) by transducing them with Brn2, Ascl1, Myt1l and NeuroD1 [204]. These induced neurons (iNs), exhibited aberrant APP processing and localization, paired with increased Aβ production, when compared to those derived from non-demented controls. Using cells from AD patients to model the disease may offer unique insights into how AD neurons function abnormally, or how they might be more vulnerable to certain environmental factors associated with AD etiology. An extension of this approach has led to the generation of induced neural progenitor-like cells (iNPCs), which might have enhanced potential for practical applications to treat neurodegenerative disorders [205]. Nevertheless, it is important to keep in mind the limitations of these models, as they might fail to recapitulate, or lose during their generation, epigenetic aberrations that are potentially crucial for disease onset and progression and that may be induced by culture conditions, unknown environmental or age-related factors.

In conclusion, in the future, animal models of familial and specifically of sporadic AD, such as the anti-nerve growth factor (AD11) transgenic mice [206] may benefit from incorporating some of the key concepts demonstrated in the literature reviewed in this article, specifically considering the plethora of epigenetic changes and changes in expression of components of the cellular epigenetic machinery associated with AD we discussed. In particular, epigenetic changes are of crucial importance in adult NSCs, and the incorporation of information regarding epigenetic changes in current AD models could advance our understanding of the potential role of NSCs and adult hippocampal neurogenesis in the pathophysiology of AD.

## Abbreviations

5-caC: 5-carboxylcytosine; 5-fC: 5-formylcytosine; 5-hmC: 5-hydroxymethylcytosine; 5-mC: 5-methylcytosine; Aβ: β-amyloid; ADAM: A disintegrin and metalloproteinase; GSK-3β: Glycogen synthase kinase-3β; AICDA: Activation-induced cytidine deaminase; APOBEC: Apolipoprotein B mRNA editing enzyme, catalytic polypeptide-like protein; AraC: Arabinofuranosyl Cytidine; AZA: 5-azacytidine; bHLH: Basic helix-loop-helix; BrdU: 5-bromo-2′-deoxyuridine; ChIP: Chromatin immunoprecipitation; CMV-GFP: Cytomegalovirus-green fluorescent protein; DAC: 5-aza-2′-deoxycytidine; DCX: Doublecortin; DG: Dentate gyrus; Dlx2: Distal-less homeobox 2; DNMT: DNA methyltransferase; DNA MeDIP: DNA immunoprecipitation; EGF: Epidermal growth factor; EGFR: Epidermal growth factor receptor; ESC: Embryonic stem cell; FGF2: Fibroblast growth factor 2; FGFR: Fibroblast growth factor receptor; Fzd: Frizzled; Gadd45: Growth arrest and DNA-damage-inducible 45; GCL: Granule cell layer; GFAP: Glial fibrillary acidic protein; GSK3β: Glycogen synthase kinase 3β; HAT: Histone acetyl transferase; HDAC: Histone de-acetylase; HMT: Histone methyltransferase; HDM: Histone demethylase; H2A: Histone 2A; H2B: Histone 2B; H3: Histone 3; H3K27me3: H3K27 tri-methylation; H3K4me3: H3K4 tri-methylation; H4: Histone 4; iPSC: Induced pluripotent stem cell; iN: Induced neuron; iNPC: Induced neural progenitor-like cell; KLF4: Krüppel-like factor 4; LEF/TCF: Lymphoid enhancer binding factor/T-cell-specific transcription factor; LTP: Long-term potentiation; MAML: Mastermind-like 1; MBD: Methyl-CpG binding domain; MCAO: Middle cerebral artery occlusion; MeCP2: Methyl-CpG-binding protein 2; MEDIP: Methylated DNA Immunoprecipitation; MiRNA: MicroRNA; Mll1: Mixed-lineage leukemia 1 protein; NaB: Sodium butyrate; NFT: Neurofibrillary tangle; NICD: Notch intracellular domain; NPC: Neural progenitor cell; NSC: Neural stem cell; OB: Olfactory bulb; OCT4: Octamer-binding transcription factor 4; PcG: Polycomb-group; PTBP2: Polypyrimidine tract binding protein 2; Ptc: Patched; Qkf: Querkopf; RAN: Ras-related nuclear protein; RBP-J: Recombination signal binding protein for immunoglobulin kappa J region; RMS: Rostral migratory stream; SEZ: Subependymal zone; SGZ: Subgranular zone; Shh: Sonic hedgehog; SiRNA: small interfering RNA; Smo: Smoothened; Sox2: Sex determining region Y (SRY)-box 2; SPT: Serine palmitoyltransferase; SVZ: Subventricular zone; SAHA: Suberoylanilide hydroxamic acid; TET: Ten-eleven translocation; TrxG: Trithorax-group; TSA: Trichostatin-A; VPA: Valproic acid.

## Competing interests

The authors declare that they have no competing interests.

## Authors’ contributions

CPF, EB, DLHH, BPFR designed the manuscript, and all authors were involved in writing the manuscript. All authors read and approved the final manuscript.
